# A systematic review of instruments for measuring outcomes in economic evaluation within aged care

**DOI:** 10.1186/s12955-015-0372-8

**Published:** 2015-11-09

**Authors:** Norma B. Bulamu, Billingsley Kaambwa, Julie Ratcliffe

**Affiliations:** Flinders Health Economics Group, School of Medicine, Flinders University, A Block, Repatriation General Hospital, 202-16 Daws Road, Daw Park, SA 5041 Australia

**Keywords:** Quality of life, Older people, Aged care, Preference-based instruments, Economic evaluation

## Abstract

**Background:**

This paper describes the methods and results of a systematic review to identify instruments used to measure quality of life outcomes in older people. The primary focus of the review was to identify instruments suitable for application with older people within economic evaluations conducted in the aged care sector.

**Methods:**

Online databases searched were PubMed, Medline, Scopus, and Web of Science, PsycInfo, CINAHL, Embase and Informit. Studies that met the following criteria were included: 1) study population exclusively above 65 years of age 2) measured health status, health related quality of life or quality of life outcomes more broadly through use of an instrument developed for this purpose, 3) used a generic preference based instrument or an older person specific preference based or non-preference based instrument or both, and 4) published in journals in the English language after 2000.

**Results:**

The most commonly applied generic preference based instrument in both the community and residential aged care context was the EuroQol - 5 Dimensions (EQ-5D), followed by the Adult Social Care Outcomes Toolkit (ASCOT) and the Health Utilities Index (HUI2/3). The most widely applied older person specific instrument was the ICEpop CAPability measure for Older people (ICECAP-O) in both community and residential aged care.

**Conclusion:**

In the absence of an ideal instrument for incorporating into economic evaluations in the aged care sector, this review recommends the use of a generic preference based measure of health related quality of life such as the EQ-5D to obtain quality adjusted life years, in combination with an instrument that has a broader quality of life focus like the ASCOT, which was designed specifically for evaluating interventions in social care or the ICECAP-O, a capability measure for older people.

**Electronic supplementary material:**

The online version of this article (doi:10.1186/s12955-015-0372-8) contains supplementary material, which is available to authorized users.

**A systematic review of outcome measures for economic evaluation in aged care**

## Background

In the year 2000, 11 % (605 million) of the world’s population was over 60 years of age and this figure is forecast to rise to 21 % (2 billion persons) by 2050 [1]. The fastest growing cohort in this population is the oldest old (over 80 years of age) accounting for 14 % of the older people population in 2014 and expected to increases to over 19 % by 2050 [1]. Older people currently represent the fastest growing age group in most developed countries and are major users of health and aged care services. Aged care services have an important role to play in enhancing the self-worth, independence and quality of life of older people [2, 3]. The projected exponential increase in the prevalence of older people living in the community with cognitive decline, frailty and other co-morbidities will inevitably contribute to a significant increase in the demand for, and utilization of aged care services in the future [1]. Most governments in developed countries subsidise different types of aged care services to enable older people to remain living at home including home care packages to support activities of daily living, nursing care, meals services, adult day care services, equipment and home adaptations, re-ablement services (to assist older people in recovering from and adapting to physical and mental illness) and support for people living with dementia [4–7].

In measuring the impact of service innovations in aged care, researchers in health economics and other disciplines are increasingly recognising that quality of life is a multi-dimensional concept and the impact of interventions for older people goes beyond health status, incorporating psychosocial and emotional well-being, independence, personal beliefs, material well-being and the external environment that influences development and activity [8–10]. Older people’s interpretation of quality of life is based on their capability to achieve those things or participate in activities they value, viewing health as a resource to facilitate their participation in activities of daily living and social interactions [10–14]. The value they obtain from health care services and other interventions goes beyond physical functioning or the health dimensions, as measured by health related quality of life (HRQOL) instruments, to include non-health dimensions such as security in their physical environment, independence, sense of value and attachment, which are only captured by broader instruments [15, 16]. It is therefore important that instruments used to measure and value quality of life outcomes in the aged care sector capture such broader quality of life outcomes.

Instruments for measuring health status and/or quality of life may be differentiated into preference based and non-preference based. Preference based instruments typically incorporate scoring algorithms which are based upon the preferences of a general population sample for the health and/or quality of life states defined by the instrument elicited using one or more valuation methods such as the visual analogue scale (VAS), time trade off (TTO), standard gamble (SG) and discrete choice experiments (DCE) [17, 18]. Preference based instruments are typically used by health economists and health service researchers within economic evaluations in a cost utility analysis framework (CUA) where the main measure of outcome is quality adjusted life years (QALYs). Non-preference based instruments are not suitable for application in CUA because they do not facilitate the calculation of QALYs. Table 1 summarises some of the most popular generic preference based and generic non-preference based instruments.

**Table 1:** Generic preference based and non-preference based instruments

Instrument	Dimensions	Levels	Valuation	Source of preference weights
Generic preference based instruments				
EuroQol - 5 Dimensions (EQ-5D)^a^	Mobility, self-care, usual activities, pain/discomfort and anxiety/depression [97]	3–5	TTO and VAS	Adult general population samples from a number of countries including Australia, UK, USA, Spain, Netherlands, Denmark, France, Germany, Japan, Thailand and Zimbabwe
Health Utilities Index Mark2 (HUI2)	Sensation, mobility, emotion, cognition, self-care, pain and fertility [98]	3–5	VAS and SG	Canada (age groups 5–37, 12–16, 8–16), USA (18–89), Australia (15+), UK (general population) and Uruguay (8–17 age group)
Health Utilities Index Mark3 (HUI3)	Vision, hearing, speech, ambulation, dexterity, emotion, cognition and pain [98]	5–6	VAS	
Short Form 6 Dimensions (SF-6D)	Physical functioning, role limitation, social functioning, pain, mental health and vitality [99]	4–6	SG	Adult general population samples from UK, Japan, Hong Kong, Australia, Brazil
Assessment of Quality of Life instrument (AQOL)^b^	Independent living, happiness, mental health, coping, relationships, self-worth, pain and senses [100]	4–6	TTO	Australia (adult general population sample)
Quality of Wellbeing scale (QWB)	Mobility, physical activity and social activity [101]	Multi-level	VAS	USA (adult general population sample)
15-Dimensions (15D)	Health-mobility, vision, hearing, breathing, sleeping, eating, speech, elimination, usual activities, mental function, discomfort and symptoms, depression, distress, vitality and sexual activity [102]	4–5	VAS	Finland (adult general population sample)
Adult Social Care Outcomes Toolkit (ASCOT)^c^	Personal cleanliness and comfort, Accommodation cleanliness and comfort, Food and drink, Safety, Social participation and involvement, Occupation, Control over daily life and Dignity [19, 20]	3	BWS and TTO	UK (adult general population sample)
Generic non-preference based instruments				
Short Form 36 (SF-36)	8 attributes; physical functioning (10 items), Role - physical (4 items), Bodily pain (2 items), General health (5 items), Vitality (4 items), Social functioning (2 items), Role - emotional (3 items) and Mental health (5 items) [103]	2–6	N/A	N/A
Short Form 12 (SF-12)	Physical functioning (2 items), role limitations (2 items), bodily pain (1 item), general health perceptions (1 item), vitality (1 item), social functioning (1 item), role limitations because of emotional problems (2 items), general mental health or psychological distress and psychological well-being (2 items) [103]	3-6	N/A	N/A
World Health Organisation Quality of Life brief instrument (WHOQoL-Bref)	Physical characteristics, Psychological aspects, Social relationships and Environmental circumstances [104]	6	N/A	N/A
Nottingham Health Profile (NHP)	Health; energy, pain, emotional reactions, sleep, social isolation, and physical mobility and General life; occupation, housework, social life, family life, sexual function, hobbies and holidays [105]	2	N/A	N/A

^a^A new 5 level version of the EQ-5D was launched in January 2014 [106]

^b^Four instruments based on the number of dimensions: AQOL-4D, -6D, -7D and -8D [100]

^c^Other versions of ASCOT are SCT4 (a four-level self-report version for use in community settings), INT4 (a four-level interview version used in community settings) and the CH3 (a three-level rating schedule based on observation and interviews used in communal living settings) [20]

In contrast to generic preference based instruments, condition specific and population specific preference based instruments focus upon one condition or disease area or population of interest. Population specific preference based instruments have been designed to be utilised with a single population group e.g. children or older people. Examples of population specific preference based instruments include the Adult Social Care Outcomes Toolkit (ASCOT) [19, 20] designed to measure quality of life for individuals receiving social care and the older person specific ICEpop CAPability measure for Older people (ICECAP-O), a measure of capability [12]. Table 2 summarises some of the most popular older person specific instruments.

**Table 2:** Older people specific instruments

Instrument	Dimensions	Levels	Scoring algorithm
Preference based			
ICEpop CAPability measure for Older people (ICECAP-O)	Attachment, Security, Role, Enjoyment and Control [92]	4	UK
Non-preference based			
Older People’s Quality of Life (OPQOL)^a^	Life overall, health, social relationships and participation, independence, control over life, freedom, and area: home and neighbourhood, psychological and emotional wellbeing, financial circumstances, and religion/culture [89]	5	N/A
Control Autonomy Self-realization and Pleasure (CASP 19)	Control, Autonomy, Self-realization and Pleasure [107]	4	N/A
World Health Organisation Quality of Life Instrument-Older Adults Module (WHOQoL-Old)^b^	Sensory functioning, autonomy, past-present-future activities, social participation, death and intimacy [108]	5	N/A

^a^A shorter 13 item version the OPQOL-Brief has been developed

^b^Three shorter versions of the WHOQoL-Old have been developed, each having only 6 items, one item per domain, as opposed to the original 24 items

This paper describes the methods and results of a systematic review to identify instruments that have been used in measuring quality of life outcomes in older people and documents the contexts in which the instruments have been applied. The primary focus is on instruments suitable for application within a CUA in the aged care sector. The findings from this review will be utilised to inform the design of economic evaluations in community aged care service delivery in Australia. The findings also have wider applicability internationally for researchers designing and conducting economic evaluations with dependent older people in determining the most suitable instruments for application in the aged care sector.

The focus on older-people-specific outcomes is motivated by assertions in the literature that economic evaluations of interventions aimed at older people should be conducted using outcome measures tailored towards meeting the goals of services consumed by older people [16]. Research also indicates that such measures need to capture broader quality of life outcomes such as affection and control that extend beyond health related quality of life whilst also recognising that many older people view health as a resource to facilitate their participation in activities of daily living and social interactions [10, 11, 15]. Different age groups of the population have also been reported to prioritize different areas of life as important, with older people being the most likely to prioritize their health and ability to get out while younger people are more likely to prioritize work, finances, having chances to learn new skills and their sex lives [21–23].

A recent systematic review conducted by Makai and colleagues [15] identified instruments suitable for applications in economic evaluations of interventions in older people receiving long term care. This systematic review builds upon the work previously conducted in two main ways; firstly by capturing the three year period beyond their systematic review and secondly by focusing in more detail upon the particular contexts in which the instrument/s were applied.

Capturing information on and gaining insights into the context within which instruments have been previously applied is particularly relevant in guiding the selection of the most appropriate instrument/s for application within economic evaluation in the aged care sector [24]. Some quality of life instruments have been specifically developed for use within certain settings. For instance, the developers of the ASCOT originally designed the instrument to capture information about an individual’s social-care-related quality of life within community and residential settings [19]. It could also be argued that health-related quality of life instruments such as the EuroQoL 5 dimensions (EQ-5D) are more suitable for individuals receiving health-focussed interventions such as those in hospital where the primary objective is the maintenance of or improvement in health. There is evidence to indicate that there are differences in quality of life perceptions between hospitalised/ambulatory and non-hospitalised older adults [23]. Fassino et al. [25] also showed that aspects of quality of life that matter to dependent older people (individuals dependent on others for their day-to-day living) differ from those that matter to independent older people and a study by Bowling et al. [26] found that better functional ability was related to better quality of life in older age. Bowling et al. [27] also postulated that the multifaceted nature of independence, particularly in older age, is mostly ignored in the wider quality of life measurement literature.

In this study, therefore, we sought to assess whether quality of life instrument use differed according to context, which was defined by the location or setting in which services for older people were being provided, i.e. in the community, residential facilities or within a hospital and according to the level of dependency for older people living in the community (specifically whether the study population was made up of dependent or independent older people). For the purposes of this review, dependency was defined as frailty or individuals dependent in activities of daily living as assessed by instruments such as the Barthel index and individuals who required or lived with an informal carer such as older people with cognitive impairment and those who have experienced or are recovering from stroke. Studies where majority of the study population was comprised of dependent older people were classified under the dependent heading.

Therefore, this paper seeks to provide arguments for the suitability (or otherwise) of the different instruments in economic evaluations of interventions for older people in various contexts within the aged care sector.

Specifically, the three main objectives of the review were:

- To identify instruments used in the published literature to measure quality of life outcomes for older people
- To identify the different contexts in which the instruments have been used
- To provide arguments for the appropriateness and suitability of the different quality of life instruments within a cost utility analysis (CUA) framework of service delivery innovations in aged care.

## Review

### Methods

The key search questions to be answered by this review were consistent with the three main objectives previously specified. The review process was consistent with the PRISMA guidelines for the conduct of systematic reviews [28].

### Databases

PubMed, Medline, CINAHL, Scopus, and Embase, PsycInfo, informit and Web of science.

### Search terms

Keywords were replicated based on the review undertaken by Makai et al. [15] with the addition of two more concepts (instruments and the contexts in which quality of life was measured) as well as appropriate subject headings and keywords based on the objectives of this review. Five major concepts were applied in this search; quality of life, the population (older people aged 65 years and over), validity, instruments and study contexts defined as community aged care or residential aged care. These concepts were combined with the ‘AND’ operator. The full search strategy, including subject headings and key words, used in Medline is attached in Additional file 1. The same broad strategy was replicated in other databases with appropriate adjustments made to align the strategy to the requirements of these other databases.

### Selection criteria

Studies that met the following criteria were considered: 1) measured quality of life and/or health status and/or health related quality of life as a primary or secondary outcome, either as a snapshot/cross-sectional or longitudinally over time in aged care settings, 2) used a generic or older person specific preference based instrument or a non-preference based older person specific quality of life instrument or both 3) study population was exclusively 65 years and over, dependent older people living in the community or in residential aged care facilities, and 4) published in peer reviewed journals in the English language between 2000 and July 2015.

Studies were excluded if 1) study population was not exclusive to people aged 65 years and over 2) study population was focused primarily upon patients in the health system and/or not comprised of dependent older people living in the community or in residential aged care facilities 3) only disease specific or generic non-preference based measures of quality of life/health related quality of life were used and studies in which quality of life was not measured using an instrument or they used questionnaires specifically designed for the study, 4) dissertations, commentaries, conference papers or review articles and studies for which the full text article could not be obtained.

To assess the reliability of the study selection process, selection was performed by all three authors on a random sample of 5 % of the studies by using the selection criteria described above. The overall agreement was then calculated using Cohen’s kappa statistic [26].

## Results

### Study selection process

**Fig. 1:**
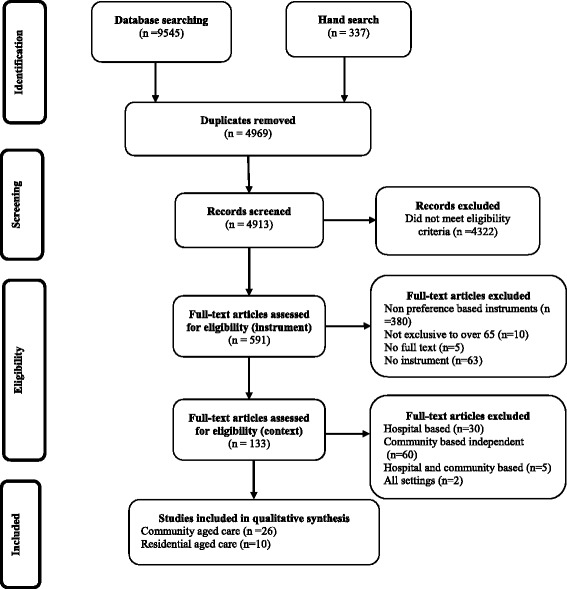
Study selection process

Figure 1 presents the study selection process which was divided into four key stages:

i) Identification: In July 2015, 9545 studies were identified from the online databases and an additional 337 studies from backward/forward searches and basic internet search using the key words. 4969 studies were eliminated because they were found to be duplicates.
ii) Screening: 4913 titles and abstracts were screened for eligibility. 4322 studies were excluded as they did not meet the eligibility criteria.
iii) Eligibility: 591 full texts articles were assessed at this stage. All three authors independently assessed 5 % of the identified studies and overall agreement was then calculated using Cohen’s kappa statistic [28] 380 studies were excluded because they measured quality of life using generic non-preference based instruments while 63 studies used questionnaires specifically designed for the study, the study population in 10 studies was not exclusive to people over 65 years of age and the full text articles could not be obtained for five studies. A further 97 studies were eliminated whose study population was focused primarily upon patients in the health system and/or not comprised of dependent older people living in the community or in residential aged care facilities.
iv) Included: 36 studies were considered in the qualitative synthesis; 26 studies undertaken among dependent older people receiving community aged care services and 10 studies among those residing in aged care facilities. The chance-corrected agreement between the abstracts selected by the primary author and the two co-authors was in the range of 0.77 and 0.88, with an average kappa statistic of 0.81, which was substantial/almost perfect [29].

### Key finding 1: study characteristics

Full reports of the included studies were read to extract information relating to the population and country of the research, sample size and type of study, the instrument used and the context in which the instrument/s were applied. *Details of all the studies assessed for eligibility and classified by context in this review are provided in* Table 3*.*

**Table 3:** Classification of studies by context

Title	Population	Instruments used	Main findings
Community-based dependent older people			
Ability to perform activities of daily living is the main factor affecting quality of life in patients with dementia [37]	Denmark	EQ-5D	Dependency upon others to perform ADL was the main factor affecting HRQL
	N = 244		
An assessment of the construct validity of the ASCOT measure of social care-related quality of life with older people [30]	UK	ASCOT	This study provides some evidence for the construct validity of the ASCOT attributes and therefore support for ASCOT’s use in economic evaluation and demonstrated the feasibility of its use among older people
	N = 301		
Comorbid psychosocial symptoms and quality of life in patients with dementia [38]	USA	EQ-5D *(patient and proxy)*	Authors discuss the psychometric and conceptual implications of possible differences between self- and other-ratings of quality of life, and treatment implications for caregiver-education interventions
	N = 89 pairs		
Comparing measurement properties of the EQ-5D-3 L, ICECAP-O and ASCOT in frail older people [24]	Netherlands	EQ-5D	Our findings support the adoption of ICECAP-O and ASCOT as outcome measures in economic evaluations of care interventions for older adults that have a broader aim than health-related QOL
	N = 190	ICECAP-O	
		ASCOT	
Day care centre attendance and quality of life in depressed older adults living in the community [53]	Italy	EQ-5D	Therefore, in older outpatients suffering from a depressive disorder without dementia the attendance of a DC was an independent correlate of the quality of life
	N = 149		
Depressive symptoms and cognitive status affect health-related quality of life in older patients with Parkinson’s disease [39]	USA	EQ-5D	Patients with PD had significantly higher (worse) GDS scores than matched controls and were more likely to take antidepressant medication
	N = 101		
Dimensions and correlates of quality of life according to frailty status: a cross-sectional study on community-dwelling older adults referred to an outpatient geriatric service in Italy [47]	Italy	OPQOL	Five of the seven dimensions of quality of life were negatively affected by frailty, but only one SOF criterion for frailty (reduced energy level) was independently related to quality of life after correction for age, functional status and depression
	N = 239		
Dutch translation and cross-cultural validation of the Adult Social Care Outcomes Toolkit (ASCOT) [48]	Netherlands	ASCOT	This study provides preliminary evidence that the Dutch translation of the ASCOT is valid, reliable and comparable to the original English version
	N = 190		
Exploration of the content validity and feasibility of the EQ-5D-3 L, ICECAP-O and ASCOT in older adults [49]	Netherlands	EQ-5D	Researchers who intend to use the EQ-5D, ICECAP-O or ASCOT in economic evaluations of care services for older adults, should be aware of the response issues that occur during the administration of these measures
	N = 10	ICECAP-O	
		ASCOT	
Factors Related to Performance-Based Mobility and Self-reported Physical Activity in Individuals 1-3 Years after Stroke: A Cross-sectional Cohort Study [54]	Sweden	EQ-5D	Individuals perceived disabilities that are partly potentially modifiable 1-3 years after stroke
	N = 195		
Health utility scores in Alzheimer’s disease: differences based on calculation with American and Canadian preference weights [40]	Canada	EQ-5D	In AD studies, researchers should calculate health utility scores by using preference weights obtained in the general population of their country of interest
	N = 216		
Health-related quality of life in Parkinson disease: correlation between Health Utilities Index III and Unified Parkinson’s Disease Rating Scale (UPDRS) in U.S. male veterans [41]	Canada	HUI3	Poor self-care in PD reflected by worsening UPDRS-II scores is strongly correlated with low generic HRQL
	N = 68		
Loneliness and Quality of Life in Chronically Ill Rural Older Adults [50]	USA	CASP-12	Nurses should assess for loneliness as part of their comprehensive assessment of patients with chronic illness
	N = 60		
Measuring health status and decline in at-risk seniors residing in the community using the Health Utilities Index Mark 2 [51]	Canada	HUI2	The HUI2 measure of HRQL in older persons at risk for institutionalization appears to reflect health status at a point in time and to be responsive to changes in health status over time
	N = 192		
Measuring the outcomes of long-term care [31]	UK	ASCOT	The results support our hypothesis that ASCOT has greater construct validity in this case
	N = 224	EQ-5D	
Older People’s Quality of Life (OPQOL) scores and adverse health outcomes at a one-year follow-up. A prospective cohort study on older outpatients living in the community in Italy [52]	Italy	OPQOL	In an older outpatient population in Italy the OPQOL total score and its health-related sub-score were independent predictors of several adverse health outcomes at one year
	N = 239		
Predictors of Family Caregiver Ratings of Patient Quality of Life in Alzheimer Disease: Cross-Sectional Results from the Canadian Alzheimer’s Disease Quality of Life Study [42]	Canada	EQ-5D	Caregiver ratings of patient function and depression were consistent independent predictors of caregiver-rated quality of life, using a spectrum of quality of life measures, while measures of patient cognition and caregiver burden and depression were not
	N = 412	QWB	
		HUI3	
		SF-36	
Predictors of Patient Self-Ratings of Quality of Life in Alzheimer Disease: Cross-Sectional Results From the Canadian Alzheimer's Disease Quality of Life Study [43]	Canada	EQ-5D	Self-rated symptoms of depression were a consistent independent predictor of patient-rated quality of life across diverse quality of life measures, while performance-based measures of cognition and informant-based functional status were not
	N = 370	QWB	
Predictors of quality of life in older people living at home and in institutions [32]	Poland	EQ-5D	The relative contribution of functional and medical comorbidities, as well as health-promoting behaviors to quality of life, may be different in community-dwelling and institutionalized elders
	N = 312		
Psychometric properties of the EQ-5D in a study of people with mild to moderate dementia [44]	Germany	EQ-5D	The study showed that the EQ-5D is especially applicable to patients with mild dementia and their caregivers as proxies
	N = 390		
Responsiveness and construct validity of the health utilities index in patients with dementia [45]	USA	HUI3	Our results support the construct validity of the proxy-rated HUI2/3 in patients with moderate to severe dementia. The proxy-rated HUI should be used in patients with moderate to severe dementia, but the self-rated HUI may be appropriate for subjects with milder cognitive impairment
	N = 408	*(proxy completed)*	
Sex differences in the relative contribution of social and clinical factors to the Health Utilities Index Mark 2 measure of health-related quality of life in older home care clients [33]	Canada and USA	HUI2	For females and males, HRQL scores were negatively associated with conditions predictive or indicative of disability and with markers of psychosocial stress
	N = 514		
Telephone reliability of the French Activity Index and EQ-5D amongst older adults [34]	Australia	EQ-5D	Telephone administration of the FAI and EQ-5D instruments provides comparable results to face-to-face administration amongst older adults deemed to have cognitive functioning intact at a basic level, indicating that this is a suitable alternate approach for collection of this information
	N = 53		
A validation of the ICECAP-O in a population of post-hospitalized older people in the Netherlands [35]	Netherlands	ICECAP-O	The ICECAP-O seems to be a valid instrument of capability-wellbeing in older, post-hospitalized people, showing good convergent validity with health and wellbeing instruments, and is able to discriminate between elderly with various health profiles
	N = 296	EQ-5D	
Validation study of the prototype of a disease-specific index measure for health-related quality of life in dementia [46]	Netherlands	EQ-5D	The DQI prototype proved valid and feasible for patients and caregivers and is appropriate for very mild to moderate dementia
	N = 145		
What can local authorities do to improve the social care-related quality of life of older adults living at home? Evidence from the Adult Social Care Survey [36]	UK	ASCOT	SCRQoL is significantly lower for older adults who find it more difficult to find information and advice, for those who report that their home design is inappropriate for their needs and for those who find it more difficult to get around their local area
	N = 29935		
Residential facility-based older people			
Capabilities and quality of life in Dutch psycho-geriatric nursing homes: an exploratory study using a proxy version of the ICECAP-O [55]	Netherlands	ICECAP-O	ICECAP-O measures a more general concept than health-related quality of life and can differentiate between restrained and non-restrained psycho-geriatric clients
	N = 122	EQ-5D	
Determinants of health-related quality of life in institutionalized older persons in northern Sydney [58]	Australia	EQ-5D	Common health states that may cause loss of independence and dignity are strongly, and independently, associated with the HRQL of institutionalized older persons
	N = 612		
Effects of cognitive stimulation therapy Japanese version (CST-J) for people with dementia: A single-blind, controlled clinical trial [56]	Japan	EQ-5D	The CST-J shows promising improvements in cognition, mood, and aspects of quality of life for people with dementia in Japanese care settings
	N = 56		
Exercise for depression in care home residents: a randomised controlled trial with cost-effectiveness analysis (OPERA) [64]	UK	EQ-5D	The results do not support the use of a whole-home physical activity and moderate-intensity exercise programme to reduce depression in care home residents
	N = 1054		
Performance of the EQ-5D and the EQ-5D + C in elderly patients with cognitive impairments [57]	Netherlands	EQ-5D	We conclude that the EQ-5D performs well for evaluating HRQL in a population with cognitive impairments
	N = 196		
Quality of life of older frail persons receiving a post-discharge program [59]	Australia	ICECAP-O	The type and intensity of programs offered to older frail people post hospital admission can impact on their recovery and quality of life gains
	N = 351	EQ-5D	
Quality of life outcomes for residents and quality ratings of care homes: is there a relationship? [60]	UK	ASCOT	The approach to providing quality ratings by the regulator in England is currently under review. Future quality indicators need to demonstrate their relationship with quality of life outcomes if they are to be a reliable guide to commissioners and private individuals purchasing care
	N = 366		
Quality of life and attitudes to ageing in Turkish older adults at old people’s homes [61]	Turkey	WHOQoL-Old	The results indicated that there was significant relationship between QOL and attitudes to ageing of older adults
	N = 120		
Strategies to implement community guidelines on nutrition and their long tern clinical effects in nursing home residents [62]	Sweden	EQ-5D	An extended model of implementation of nutritional guidelines, including guidance and feedback to NH staff, did not affect nutritional status but may be associated with a delayed cognitive decline in communicative NH residents
	N = 101		
The agreement between proxy and self-completed EQ-5D for care home residents was better for index scores than individual domains [63]	UK	EQ-5D *(proxy respondent)*	Proxies appear to be an acceptable source of data for index scores and QALYs but may be less reliable if individual domains are considered
	N = 565 pairs		
Community based independent older people			
A comparison of the ICECAP-O with EQ-5D in a falls prevention clinical setting: are they complements or substitutes? [11]	Canada	ICECAP-O	Our study suggests that the EQ-5D and ICECAP-O provide complementary information
	N = 215	EQ-5D	
A concise alternative for researching health-related quality of life in older people [109]	Wales	EQ-5D	The EQ-5D may provide a valid measure of health-related quality of life in a cross-sectional population sample of older adults, although the emphasis of the scale is very much on physical health and functioning
	N = 423	SF-36	
A cross-sectional study of quality of life in an elderly population (75 years and over) with atrial fibrillation: secondary analysis of data from the Birmingham Atrial Fibrillation Treatment of the Aged study [110]	UK	EQ-5D	In the absence of co-morbidity, chronic AF has little impact on generic quality of life in an elderly non-acutely ill population
	N = 1762	SF-12	
A measure of quality of life in early old age: The theory, development and properties of a needs satisfaction model (CASP-19) [107]	UK	CASP-19	The CASP-19 appears to be a useful scale for measuring quality of life in older people
	N = 286		
A short measure of quality of life in older age: the performance of the brief Older People's Quality of Life questionnaire (OPQOL-brief) [111]	UK	OPQOL, CASP-19 and the WHOQoL-OLD	The OPQOL-brief is of value in assessment of interventions where a rigorously tested, short measure is required
	N = 589		
An assessment of the construct validity of the descriptive system for the ICECAP capability measure for older people [16]	UK	ICECAP-O	This study provides some early evidence for the construct validity of the ICECAP measure
	N = 315		
An assessment of the relationship between informal caring and quality of life in older community-dwelling adults – more positives than negatives? [112]	Australia	ICECAP-O	A caring role is associated with a relatively high quality of life, that is comparable to that experienced by older people who categorize themselves in a non-caring role
	N = 786		
Assessing Quality of Life among British Older People Using the ICEPOP CAPability (ICECAP-O) Measure [92]	UK	ICECAP-O	Distribution of ICECAP-O values by electoral ward enabled the identification of areas of deprivation, although the associations were strong only for enjoyment and control
	N = 809		
Assessing quality of life in the elderly: A direct comparison of the EQ-5D and AQoL [80]	UK	EQ-5D	Although the AQoL appeared to have more favourable construct validity, the EQ-5D was easier to administer, had a higher completion rate, and appeared more sensitive to change
	N = 145	AQoL	
Cognition, daily living, and health-related quality of life in 85-year-olds in Sweden [113]	Sweden	EQ-5D	Cognitive impairment is associated with reduced quality of life
	N = 373		
Developing capacities in aging studies in the Middle East: Implementation of an Arabic version of the CANE IV among community-dwelling older adults in Lebanon [114]	Lebanon	EQ-5D	The Arabic version of the CANE appears acceptable in assessing needs of older adults in South Lebanon
	N = 322		
Development and measurement properties of the self-assessment version of the INTERMED for the elderly to assess case complexity [115]	Netherlands	EQ-5D and SF-36	This study supports the feasibility, reliability and validity of the IM-E-SA
	N = 338		
Development of the Japanese 15D instrument of health-related quality of life: verification of reliability and validity among elderly people [116]	Japan	15D	The Japanese version of the 15D showed sufficient internal consistency and moderate repeatability
	N = 430		
Drug treatment in the elderly: an intervention in primary care to enhance prescription quality and quality of life [117]	Sweden	EQ-5D	The intervention seems to have had no effect on quality of prescriptions or quality of life
	N = 150		
Effect of preventive primary care outreach on health related quality of life among older adults at risk of functional decline: a randomized controlled trial [118]	Canada	HUI3	The results of this study do not support adoption of this preventive primary care intervention for this target population of high risk older adults
	N = 719		
Effects of risk-based multifactorial fall prevention on health-related quality of life among the community-dwelling aged: a randomized controlled trial [119]	Finland	15D	Fall prevention produced positive effects on some dimensions of HRQL with men benefiting more than women
	N = 591		
Exercise in elderly patients with chronic heart failure in primary care: Effects on physical capacity and health-related quality of life [120]	Sweden	EQ-5D	This study shows that exercise conducted in groups in primary care and in the patients' homes could be used in elderly patients with CHF
	N = 60	SF-36	
Exploration of the association between quality of life, assessed by the EQ-5D and ICECAP-O, and falls risk, cognitive function and daily function, in older adults with mobility impairments	Canada	ICECAP-O	Both the EQ-5D and ICECAP-O demonstrate associations with falls risk and general balance and mobility; however, only the ICECAP-O was associated with cognitive status among older adults with mobility impairments
	N = 215	EQ-5D	
Falls and EQ-5D rated quality of life in community-dwelling seniors with concurrent chronic diseases: a cross-sectional study [121]	Germany	EQ-5D	The findings suggest that falls are negatively associated with EQ-5D rated quality of life independent of a variety of chronic diseases and conditions
	N = 1792		
Falls-related self-efficacy is independently associated with quality-adjusted life years in older women [122]	Canada	EQ-5D	Although falls-related self-efficacy was independently associated with QALYs, there may well be other factors not investigated, such as risk taking and psychological measures, which could account for some of the association
	N = 135		
Functional status and quality of life 12 months after discharge from a medical ICU in healthy elderly patients: a prospective observational study [123]	Spain	EQ-5D	The survival rate of elderly medical patients 12 months after discharge from the ICU is low although functional status and quality of life remained similar to baseline in most of the survivors
	N = 112		
Happiness, subjective and objective oral health status, and oral health behaviors among Korean elders [124]	Korea	EQ-5D	Oral impacts which might persistently affect one’s daily life need to be considered in designing and delivering public services aimed to promote people’s happiness
	N = 479		
Health status of the advanced elderly in six European countries: results from a representative survey using EQ-5D and SF-12 [125]	Belgium, France, Germany, Italy, the Netherlands and Spain	EQ-5D and SF-12	More than two thirds of the advanced elderly report impairment of health status
	N = 1659		
Health-related quality of life measurements in elderly Canadians with osteoporosis compared to other chronic medical conditions: a population-based study from the Canadian Multicentre Osteoporosis Study (CaMos) [126]	Canada	HUI3	The decrement in HUI3 score seen in participants with osteoporosis was comparable to that observed in other chronic medical conditions, such as arthritis, COPD, diabetes mellitus or heart disease
	N = 4,550		
Impaired Health-Related Quality of Life in Elderly Women is Associated With Multimorbidity: Results From the Korean National Health and Nutrition Examination Survey [127]	Korea	EQ-5D	Both the amount and pattern of chronic diseases have been associated with quality of life in elderly populations
	N = 1419		
Independent contribution of overweight/obesity and physical inactivity to lower health-related quality of life in community-dwelling older subjects [128]	Poland	EQ-5D	Overweight/obesity and sedentary lifestyle are independent predictors of lower HRQL in community-dwelling seniors aged 66–79 years
	N = 300		
Long term effects of exercise training on physical activity level and quality of life in elderly coronary patients - a three- to six-year follow-up [129]	USA	EQ-5D	Even a short period of supervised exercise training has the potential to positively influence physical activity level for as long as three to six years
	N = 93		
Measuring quality of life in older people: reliability and validity of WHOQoL-OLD [130]	Australia	WHOQoL-Old and SF-12	Overall, the WHOQoL-OLD performed well on tests of reliability and validity
	N = 100		
Medication quality and quality of life in the elderly, a cohort study [131]	Sweden	EQ-5D	This study has shown the validity of the basic principle in prescribing: the more appropriate medication the better quality of life
	N = 150		
Metabolic syndrome and quality of life in the elderly: age and gender differences [132]	Italy	HUI3	MetS is not associated with worse HRQL in community-dwelling elderly
	N = 356		
Mobility Is a Key Predictor of Change in Well-Being Among Older Adults Who Experience Falls: Evidence From the Vancouver Falls Prevention Clinic Cohort [133]	Canada	ICECAP-O	We found that 2 valid and reliable measures of mobility interacted with sex to predict changes in well-being over time
	N = 244		
Mortality in healthy elderly patients after ICU admission [134]	Spain	EQ-5D	Healthy elderly non-elective medical patients admitted to the ICU have a high mortality rate related to premorbid quality of life
	N = 230		
Multifactorial Intervention to Reduce Falls in Older People at High Risk of Recurrent Falls A Randomized Controlled Trial [135]	Netherlands	EQ-5D and SF-12	This multifactorial fall-prevention program does not reduce falls in high-risk, cognitively intact older persons.
	N = 217		
Multi-morbidity and health-related quality of life in the older population: results from the German KORA-Age study [136]	Germany	EQ-5D	Multi-morbidity caused greater impairments in HRQL than could be expected from given conditions individually/separately
	N = 4565		
Pain, Medication Use, and Health-Related Quality of Life in Older Persons With Post-herpetic Neuralgia: Results From a Population-Based Survey [137]	USA	EQ-5D	Older persons with PHN experience longstanding, severe, and debilitating pain and poor health-related quality of life
	N = 385		
Perceived Participation and Health-Related Quality of Life in 85 Year Olds in Sweden [138]	Sweden	EQ-5D	Sufficient participation was positively associated with higher health-related quality of life, and facilitating participation is an area of interest for occupational therapists
	N = 380		
Physical activity as a mediator of the impact of chronic conditions on quality of life in older adults [139]	Canada	HUI3	Physical activity partially mediates the impact of chronic conditions on quality of life
	N = 22,432		
Physical function and perceived quality of life in older persons [140]	Italy	EQ-5D	Physical function influences quality of life in older persons
	N = 73		
Potentially Inappropriate Drug Use and Health-Related Quality of Life in the Elderly [141]	USA	EQ-5D	The results supported others showing that a significant proportion of the elderly receive care that is potentially harmful and not supported by evidence-based practice
	N = 444	SF-12	
Potentially inappropriate prescribing and adverse health outcomes in community dwelling older patients [142]	Ireland	EQ-5D	Reducing PIP in primary care may help lower the burden of ADEs, its associated health care use and costs and enhance quality of life in older patients
	N = 931		
Psychological approach to successful ageing predicts future quality of life in older adults [143]	UK	OPQOL	Successful ageing is not only about the maintenance of health, but about maximising one’s psychological resources, namely self-efficacy and resilience
	N = 287		
Psychometric evaluation of the Korean version of the Self-Efficacy for Exercise Scale for older adults [144]	South Korea	EQ-5D	The SEE-K appears to have satisfactory validity and reliability among older adults in South Korea
	N = 212		
Quality of life amongst older Brazilians: A cross-cultural validation of the CASP-19 into Brazilian-Portuguese [145]	Brazil	CASP-19	In this small exploratory study the CASP-19 Brazil demonstrated good psychometric properties
	N = 87		
Quality of life and related factors: a questionnaire survey of older people living alone in Mainland China [146]	China	OPQOL	his study identified nine factors influencing the quality of life of older people living alone in Mainland China
	N = 521		
Quality of life in older outpatients living alone in the community in Italy [147]	Italy	OPQOL	Depression, having no caregiver and having never been married could provide a valuable means of identifying older people living alone who are at greater risk of a poor quality of life
	N = 239		
Quality of life related to fear of falling and hip fracture in older women: A time trade off study [148]	Australia	EQ-5D	Among older women who have exceeded average life expectancy, quality of life is profoundly threatened by falls and hip fractures
	N = 194		
Quality of well-being in older people with osteoarthritis [149]	USA	QWB	The QWB appears to be a useful and sensitive generic, utility-based measure of HRQL in people with OA
	N = 363		
Risk of malnutrition and health-related quality of life in community-living elderly men and women: The Tromsø study [150]	Norway	EQ-5D	HRQL was significantly reduced in elderly men and women at risk of malnutrition
	N = 3286		
SF-6D and EQ-5D result in widely divergent incremental cost-effectiveness ratios in a clinical trial of older women: implications for health policy decisions [151]	USA	EQ-5D and SF-6D	The incremental QALYs estimated from the SF-6D were two- to threefold greater than those estimated from the EQ-5D
	N = 155		
Sleep Apnea and Health-Related Quality of Life in African-American Elderly [152]	USA	QWB and SF-36	Sleep disturbances may impact daily living and health as much as other medical conditions
	N = 70		
Societal Consequences of Falls in the Older Population: Injuries, Healthcare Costs, and Long-Term Reduced quality of life [153]	Netherlands	EQ-5D	Fall-related injuries are age and gender related, leading to high healthcare costs, and long-term reduced quality of life
	N = 668		
Socioeconomic status and health-related quality of life among elderly people: Results from the Joint Canada/United States Survey of Health [154]	Canada and USA	HUI3	In the elderly population, HRQL was significantly associated with household income in the United States but not in Canada, controlling for socio-demographic and health indicators
	N = 1906		
The Health Consequences of Peripheral Neurological Deficits in an Elderly Cohort: An Oklahoma Physicians Resource-Research Network Study [155]	USA	HUI3	PNDs of undetermined cause, found in older patients on physical examination, appear to be associated with greater morbidity and mortality
	N = 604	QWB	
		SF-36	
The health-related quality of life and cost implications of falls in elderly women [156]	UK	EQ-5D	Interventions aimed at reducing fear of falling may produce larger gains in HRQL
	N = 11802		
The independent contribution of executive functions to health related quality of life in older women [157]	UK	EQ-5D	Our study highlights the specific executive processes of set shifting and working memory were independently associated with QALYs
	N = 135		
The influence of lower-extremity function in elderly individuals’ quality of life: an analysis of the correlation between SPPB and EQ-5D [158]	Korea	EQ-5D	An abnormal SPPB score was associated with lower quality of life
	N = 422		
The predictive value of self-rated health in the presence of subjective memory complaints on permanent nursing home placement in elderly primary care patients over 4-year follow-up [159]	Denmark	EQ-5D	Both poor SRH and SMC were associated with permanent NH placement risk among elderly primary care patients
	N = 757		
The Psychometric Properties of the Older People’s Quality of Life Questionnaire, Compared with the CASP-19 and the WHOQoL-OLD [89]	UK	OPQOL, CASP-19 and WHOQoL-OLD	The OPQOL has potential for use as a multidimensional population surveillance instrument for use with older populations, or as an outcome measure of multisector policy
	N=		
The Reliability and Validity of the Turkish Version of the World Health Organization Quality of Life Instrument-Older Adults Module (WHOQoL-Old) [160]	Turkey	WHOQoL-Old WHOQoL-Bref	The psychometric properties of the Turkish version of the WHOQoL-OLD were acceptable, indicating that the scale is reliable and valid for use with older Turkish adults (>65 years)
	N = 527		
Which measure of quality of life performs best in older age? A comparison of the OPQOL, CASP-19 and WHOQOL-OLD [161]	UK	OPQOL	The OPQOL is of potential value in the outcome assessment of health and social interventions, which can have a multidimensional impact on people’s lives
		CASP-19	
		WHOQoL-OLD	
Hospital and community-based older people			
Economic evaluation alongside a single RCT of an integrative psychotherapeutic nursing home programme [162]	Netherlands	EQ-5D	No significant differences were found on QALYs
	N = 168		
Influence of chronic cardiovascular disease and hospitalization due to this disease on quality of life of community-dwelling elderly [163]	Poland	EQ-5D	Hospitalization due to CVD results in more pronounced reduction in quality of life than CVD alone among community dwelling elderly
	N = 300		
The relationship between quality of life, health and care transition: an empirical comparison in an older post-acute population [164]	Australia	ICECAP-O	The correlations between the ICECAP-O, EQ-5D and CTM-3 instruments illustrate that capability is strongly and positively associated with health-related quality of life and the quality of care transitions
	N = 82	EQ-5D	
Elderly men’s quality of life and lower urinary tract symptoms: an intricate relationship [165]	Brazil	WHOQoL-Old	Moderate to severe LUTS are associated with worse quality of life ratings for almost all evaluation parameters
	N = 200	WHOQoL-Bref	
Quality of Life in Elderly Men With Aging Symptoms and Lower Urinary Tract Symptoms (LUTS) [166]	Brazil	WHOQoL-Old and WHOQoL-Bref	Moderate to severe ADAM and LUTS impact significantly all parameters of HRQL and generic quality of life proposed by the WHO
	N = 200		
Hospital-based older people			
Activities of Daily Living and Quality of Life of Elderly Patients After Elective Surgery for Gastric and Colorectal Cancers [167]	Japan	EQ-5D and SF-12	Of the patients 75 years old or older who underwent elective surgery for gastric or colorectal cancer, only a few showed a protracted decline in ADL and most exhibited better quality of life after surgery
	N = 232		
Cognitive, Functional, and quality of life Outcomes of Patients Aged 80 and Older Who Survived at Least 1 Year After Planned or Unplanned Surgery or Medical Intensive Care Treatment [168]	Netherlands	EQ-5D	Health-related quality of life is similar to that of an age-matched general population in the long term
	N = 204		
Costs and health outcomes of intermediate care: results from five UK case study sites [169]	UK	EQ-5D	Our work suggests a need for the development and application of robust and reliable clinical criteria for admission to IC, and close co-operation between hospital and community service providers over selection of patients and targeting of IC and acute care services to meet defined clinical need
	N = 2253		
Effectiveness of a video-based exercise programme to reduce falls and improve HRQL among older adults discharged from hospital: a pilot randomized controlled trial [170]	Australia	EQ-5D	No significant difference in self-reported quality of life and falls prevention between groups; need for further research
	N = 53	*Completed by both patient and proxy*	
Health-Related Quality of Life After Transcatheter Aortic Valve Implantation in Elderly Patients With Severe Aortic Stenosis [171]	France	EQ-5D	In high-risk patients with severe aortic stenosis, quality of life and health status improved substantially at 1 month and improvement persisted 6 months after TAVI
	N = 164		
Health-related Quality of Life among hospitalized older people awaiting residential aged care [172]	Australia	AQoL	People making the transition to residential aged care from hospital have very poor HRQL, but small gains in function seem to be related to improvement
	N = 320		
Long-term outcome of elderly patients requiring intensive care admission for abdominal pathologies: survival and quality of life [173]	Switzerland	EQ-5D	A high mortality rate and a decrease in quality of life were observed in elderly patients with severe abdominal pathologies
	N = 36	SF-36	
Multicomponent Geriatric Intervention for Elderly Inpatients With Delirium: Effects on Costs and Health-Related Quality of Life [174]	Finland	15D	Comprehensive geriatric intervention improved HRQL without increasing overall costs of care
	N = 174		
Outcome and quality of life of elderly critically ill patients: An Italian prospective observational study [175]	Italy	EQ-5D	One year after ICU discharge, medical and orthopedic patients had significantly more severe problems vis-a` -vis mobility, self-care and activity than abdominal surgical patients and control population
	N = 288		
Outcomes among older people in a post-acute inpatient rehabilitation unit [176]	Ireland	EQ-5D	Positive quality of life outcomes occurred in a range of measures in an older, frail inpatient rehabilitation population
	N = 32		
Patients undergoing sub-acute rehabilitation have accurate expectations of their health-related quality of life at discharge [177]	Australia	EQ-5D	Patients admitted for subacute in-hospital rehabilitation were able to anticipate their discharge health-related quality of life on the EQ-5D instrument with a moderate level of accuracy
	N = 232		
Proxy reporting of quality of life using the EQ-5D [178]	Canada	EQ-5D	Proxy EQ-5D responses, either for a specific point in time or for assessing change over time, may not be valid measures of self-reported quality of life among older medically-ill patients
	N = 231		
Quality of life after a sub-trochanteric fracture: A prospective cohort study on 87 elderly patients [179]	Sweden	EQ-5D	A sub-trochanteric fracture in elderly patients had a substantial negative effect on both their short and long-term HRQL
	N = 87		
Self-reported quality of life in elderly patients with aggressive non-Hodgkin’s lymphoma treated with CHOP chemotherapy [180]	Netherlands	EQ-5D	In the elderly patients the quality of life was determined by two factors, i.e. aggressiveness of disease and toxicity of treatment
	N = 132	EORTC QLQ-C30	
Specialist geriatric medical assessment for patients discharged from hospital acute assessment units: randomised controlled trial [181]	UK	ICECAP-O	This specialist geriatric medical intervention applied to an at risk population of older people attending and being discharged from acute medical units had no effect on patients' outcomes or subsequent use of secondary care or long term care
	N = 433	EQ-5D	
Systematic comprehensive geriatric assessment in elderly patients on chronic dialysis: a cross-sectional comparative and feasibility study [182]	Netherlands	EQ-6D	Older patients on chronic dialysis have a high risk of functional decline
	N = 50		
The correlation between patients, patient's relatives and healthcare professionals interpretation of quality of life - A prospective study [183]	UK	EQ-5D	Preliminary results would suggest NOK have a better perception than healthcare professionals of patient's quality of life, however doctors may be better at predicting resuscitation decisions of patients
	N = 22	SF-36	
		*(patient and proxy)*	
Two perspectives of proxy reporting of health-related quality of life using the Euroqol-5D, an investigation of agreement [184]	Australia	EQ-5D *(proxy correspondent)*	Clinician (physiotherapist) proxy-reports among this population generally had good agreement with patient self-report though this was affected by proxy perspective, patient cognition, and timing
	N = 272		
Validation of the Charlson Comorbidity Index in acutely admitted elderly patients [185]	Spain	EQ-5D	This study shows that an increased CCI predicts both short and long term mortality adequately but is not able to predict post-discharge functional decline in acutely admitted elderly patients
	N = 1313		
An investigation into the association between nutritional status and quality of life in older people admitted to hospital [186]	UK	EQ-5D	Malnutrition risk is linked to a poorer quality of life in older people on admission to hospital
	N = 149	SF-36	
Assessing Health State Utilities in Elderly Patients at Cardiovascular Risk [187]	USA	HUI3	In this large implementation of the HUI in elderly patients, the instrument did not detect any differences in estimated utilities related to having a MI
	N = 4677		
Femoral neck fractures in the elderly: Functional outcome and quality of life according to EuroQol [188]	Sweden	EQ-5D	Changes in the quality of life may be useful to identify patients who might benefit from reoperation, i.e. arthroplasty
	N = 90		
Health-related quality of life and psychological well-being in elderly patients with haemophilia [189]	Italy	WHOQoL-Old EQ-VAS and WHOQoL-BREF	Compared to age-matched controls elderly patients with haemophilia had an impaired HRQL in association with their health status
	N = 82		
Health-related quality of life in elderly patients with familial hypercholesterolemia [190]	Sweden	15D and SF-36	HRQL appears to be similar to that of age-standardized controls in the general population
	N = 37		
Internal fixation compared with total hip replacement for displaced femoral neck fractures in the elderly - A randomised, controlled trial [191]	Sweden	EQ-5D	The results of our study strongly suggest that THR provides a better outcome than IF for elderly, relatively healthy, lucid patients with a displaced fracture of the femoral neck
	N = 102		
Predictors of Improvement in Health-Related Quality of Life Among Elderly Patients With Depression [192]	USA	MOS-6 (SF-6D)	Severity of depressive symptoms at baseline was predictive of failure to improve HRQL
	N = 100		
Quality of life (QOL) among community dwelling older people in Taiwan measured by the CASP-19, an index to capture quality of life in old age [193]	Taiwan	CASP-19 and CASP-12	There was an inverse relationship between the CASP total scores and frailty, chronic diseases, depressive disorders, living alone and fall events in the past 12 months
	N = 699		
Quality of life related to fracture displacement among elderly patients with femoral neck fractures treated with internal fixation [194]	Sweden	EQ-5D	The rate of fracture healing complications and reoperations in patients with displaced fractures was high, and even in patients with uneventfully healed fractures, there was a substantial decrease in the quality of life
	N = 90		
Responsiveness of the EuroQol (EQ 5-D) and the SF-36 in elderly patients with displaced femoral neck fractures [195]	Sweden	EQ-5D	The results showed high responsiveness for both the EQ-5D and the SF-36, indicating that both instruments are suitable for use as outcome measures in clinical trials in elderly hip fracture patients
	N = 110	SF-36	
The Importance of Acuity, Stereopsis, and Contrast Sensitivity for Health-Related Quality of Life in Elderly Women with Cataracts [196]	UK	EQ-5D	Acuity, stereopsis, and contrast sensitivity each contributed to quality of life, across a range of measures, in elderly women with cataract
	N = 306		
All settings			
Whose Quality of Life Is It Anyway? The Validity and Reliability of the Quality of Life-Alzheimer's Disease (QoL-AD) Scale [25]	UK	EQ-5D	The QoL-AD has very good psychometric properties and can be completed with people with a wide range of severity of dementia
	N = 261		
Quality of life in dementia - A one-year follow-up study [19]	UK	EQ-5D	The main finding of this study is that people with dementia do not perceive that their quality of life declined over a period of one-year
	N = 60		
Valuation studies			
Valuing the ICECAP capability index for older people [12]	UK	ICECAP-O	Values that were estimated are feasible for use in practical applications of the index to measure the impact of health and social care interventions
	N = 19		

**Fig. 2:**
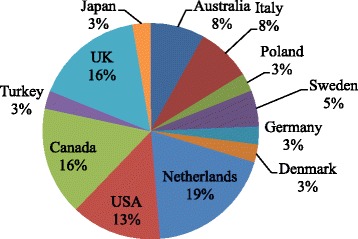
Geographical distribution of studies

Geographically studies were conducted in several countries; 7 (19 %) from the Netherlands, 6 studies (16 %) each from the UK and Canada, 5 studies (14 %) from the USA(with one study from both USA and Canada) while three studies each (8 %) were from Australia and Italy. 2 studies (5 %) were conducted in Sweden and one study (3 %) each in Poland, Germany, Denmark, Japan and Turkey. The majority of studies (59 %) were undertaken in Europe. Figure 2 is a summary of the geographical distribution of the included studies.

Twenty two studies (61 %) were undertaken using a cross-sectional design, 8 (22 %) randomised control trials, 3 (8 %) prospective cohort studies, 2 (6 %) longitudinal studies and one explorative qualitative study.

**Fig. 3:**
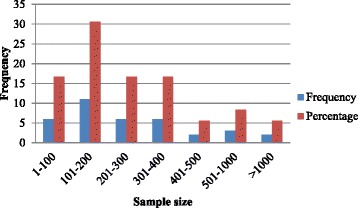
Sample size distribution

The sample sizes varied substantially from a minimum of 10 to a maximum of 29,935 older people. Figure 3 summarises the sample size distributions of included studies.

### Key finding 2: contexts and settings

The identified studies were grouped into four contexts; community based dependent, community based independent, residential facility based and hospital based older people (see Table 3). Two contexts were considered to be reflective of older people receiving aged care services; community based dependent older people and older people based in residential aged care facilities. The results reported below therefore relate to these two contexts:

- Community living dependent older people: Twenty six studies were identified; seven studies conducted in older people with no particular prevailing condition [30–36], ten studies among older people with cognitive impairment [37–46] while seven studies comprised of frail older people where cognitive status was unspecified [24, 47–52]. One study each was among older people with depression [53] and those with a previous stroke [54].
- Residential aged care context: Ten studies were identified in this context; three among residents with cognitive impairment [55–57], two studies among frail older residents where cognitive status was unspecified [58, 59] and four studies recruited samples from the general resident population who had no cognitive impairment and were not too ill to participate in the study [60–63] while one study specifically considered residents with depression [64].

### Key finding 3: instruments used to measure quality of life

Table 4 summarises the instruments that have been used to date to measure quality of life within the community and residential aged care context and Table 5 reports the frequency with which they were used.

**Table 4:** Instruments used in the identified studies

Instruments used	Number of studies		
Instrument	Community aged care (Community dependent)	Residential aged care	Total
EQ-5D	10	6	16
ASCOT	3	1	4
HUI2/3	4		4
EQ-5D + ASCOT	1		1
EQ-5D + QWB	1		1
EQ-5D + QWB + HUI3	1		1
EQ-5D + ASCOT + ICECAP-O	2		2
EQ-5D + ICECAP-O	1	2	3
CASP-12/19	1		1
OPQOL	2		2
WHOQoL-OLD		1	1
Total	26	10	36

**Table 5:** Frequency with which the different instruments were used

Instrument	Community aged care	Residential aged care	Percentage	Comment
Generic Preference based				
EQ-5D	16	7	51	Community: With ASCOT n = 1, With QWB n = 1, With QWB + HUI3 n = 1, With ASCOT + ICECAP-O n = 2, With ICECAP-O n = 1 Residential: With ICECAP-O n = 1
ASCOT	6	1	16	Community: With EQ-5D n = 1, With EQ-5D + ICECAP-O n = 2
HUI2/3	5	0	11	With EQ-5D + QWB n = 1
QWB	1		2	With EQ-5D + HUI3 n = 1
Older people specific preference based				
ICECAP-O	3	2	11	Community: With EQ-5D n = 1, With EQ-5D + ASCOT n = 2 Residential: With EQ-5D n = 1
Older people specific non preference based				
OPQOL	2	0	4	
CASP-12/CASP-19	1	0	2	
WHOQoL-Old	0	1	2	
Total	34	11	100	

In general, the most commonly applied generic preference based instrument was the EQ-5D (51 %) followed by the ASCOT (16 %), and the most widely applied older person specific instrument was the ICECAP-O (11 %) (Table 5). In the community aged care context, the most applied generic instrument was the EQ-5D (n = 16) followed by the ASCOT (n = 6) and HUI3 (n = 5) and the older people specific ICECAP-O (n = 3). Other instruments applied in this sector were the older people specific OPQOL (n = 2) and CASP-19 (n = 1) and the generic QWB (n = 1). In the residential aged care context on the other hand, the most widely applied instrument was the EQ-5D (n = 7) followed by the ICECAP-O (n = 2). Other instruments applied were the ASCOT (n = 1) and the older people specific WHOQoL-Old (n = 1).

The popularity of the EQ-5D may be attributed to several reasons including its brevity, the availability of various translations and scoring algorithms from several cultures and countries worldwide and its recommended use for the economic evaluation of new technologies by the National Institute for Health and Care Excellence (NICE) in the UK [65]. The ICECAP-O, CASP-19, OPQOL and ASCOT are all relatively newer instruments developed and validated in the UK. NICE recommends the ASCOT as the preferred measure for outcomes in social care and the ICECAP-O where outcomes in terms of capabilities are to be measured [65].

Five studies explicitly measured quality of life at more than one time point allowing some assessment of the sensitivity to change over time to be made [24, 45, 51, 63, 64]. Absolute changes in utility scores ranged from 0.003 to 0.21 based on 6-18 month follow-up periods. There is no consensus in the literature about what the minimal important difference (MID) should be (i.e. values range from 0.03 [66–68] to 0.074 [69] for changes in EQ-5D [64] and HUI3 utilities [45]). Some of the changes in quality of life reported upon in these studies were larger than the MID value reported in the literature (0.03). However, Drummond [66] indicates that if outcomes based on preference-based measures are to be used to influence resource allocation decisions, it is the difference in cost-effectiveness, such as the incremental cost per QALY, rather than the change in quality of life that is important. This therefore means that within an economic evaluation framework it is important to consider changes in quality of life in addition to the cost of bringing about such changes [66].

## Discussion

In considering the suitability of instruments for use in evaluating interventions in the aged care sector, it’s important to consider the aspects of quality of life that are most important to older people and to assess the ability (or otherwise) of each instrument to capture these aspects within the framework of economic evaluation.

This review has highlighted the multi-dimensional nature of quality of life for older people. Key elements of quality of life amongst community living dependent older people include physical and cognitive functioning [32, 34], independence in activities of daily living [37, 47], social relationships [31–33, 47], absence of morbidity or health impairments [32, 33, 51] and pyscho-social wellbeing [32, 38, 51] as well as social connectedness and accessibility within the home and community [36, 47, 50]. In the residential aged care context on the other hand key contributing factors to quality of life include independence in activities of daily living, sense of dignity and physical freedom [55, 58, 60, 70], absence of morbidity or health impairments [58] and happiness coupled with social participation [60, 61].

These findings are consistent with arguments made by several commentators that social participation [71–73], health [10, 71, 72], wealth [72–74], home and community environment [10, 72–75], and independence or control over their life [10, 72, 73] represent key dimensions for any assessment of quality of life in samples of community living dependent older people. In the residential aged care context, important dimensions highlighted in the literature include social participation in family and leisure activities [76–78], independence [76–79], peace and contentment [76, 79], security [77, 79] and spiritual well-being [77, 78].

Generic preference based instruments assess respondents’ level of physical functioning through domains such as mobility within the EQ-5D, 15D and HUI3 and independent living within the AQOL. Psychological and emotional wellbeing is accounted for by anxiety/depression on the EQ-5D and 15D, emotion on HUI3 and happiness and mental health of the AQOL. Relationships and family dimensions may be captured by the relationships domain of the AQOL-8D. However, the question remains as to whether these quality of life dimensions are interpreted in the same way by older people themselves. For example physical functioning in older people may not necessarily be linked solely to their levels of mobility but also to their ability to participate in meaningful activities that emphasise their dignity, independence and relevance to society or their significant relations [58, 71].

Of the preference based instruments identified by this review, the EQ-5D is relatively easy to administer and has a higher completion rate [49, 70, 80–82]. With consideration to respondent burden, the EQ-5D may be considered to have practical advantages as it is relatively brief with only 5 dimensions. The other generic preference based instruments have more dimensions and/or dimension levels as illustrated in Table 1 with several also having mixed response items which may be considered to impose an additional response burden. Research has however shown that the EQ-5D has higher ceiling effects when compared against other instruments such the SF-6D and this needs to be taken into account [49, 83, 84]. The recent development of the new five-level version of the EQ-5D may however minimize this ceiling effect [85, 86]. Coast et al. [87] and Hulme [88] have advocated for interviewer help to complete the instrument when used among the very elderly and those with reduced cognitive function. In fact other researchers have used proxy respondents to apply the instrument to people with mild to moderate cognitive impairment [38, 42, 63, 70]. As highlighted previously, the vast majority of preference based HRQOL instruments such as the EQ-5D were developed for application in a health care context and are narrowly focused on health status alone a dimension highlighted in both community and residential aged care contexts, but it may not be the most appropriate indicator for measuring quality of life in older people. Several researchers have argued that these instruments are unlikely to be appropriate for assessing the well-being of older people in an aged care context because broader quality of life dimensions are important in this context.

The ASCOT is a preference based measure designed to specifically measure social care related quality of life and captures dimensions of quality of life relevant to people receiving social care services [19]. The ASCOT necessarily takes a broader quality of life focus including dimensions such as dignity, safety, control over daily life and social participation which are important for older people in both community and residential aged care [24, 49]. The ASCOT may therefore be considered a relevant instrument to apply when assessing quality of life in relation to service innovations in the aged care sector.

The older person specific instruments identified by this review reflect quality of life in a broader sense and thereby tend to address the majority of key domains previously highlighted as important to older people.

The OPQOL may be considered to represent the most comprehensive older people specific instrument developed to date as it contains quality of life domains/dimensions identified as important for both community and residential aged care contexts and it incorporates both health status and broader quality of life domains [89–91]. However, the OPQOL currently has limited use in an economic evaluation framework because it is not preference based.

The ICECAP-O also encompasses quality of life dimensions that are relevant in both community and residential aged care contexts such as independence or control, security, social participation or attachment, and it has been validated for use in older people in the health and the aged care sectors in several European countries [16, 49, 55, 70, 92]. It is also notable that good construct validity has been reported for the ICECAP-O when used in older people with mild to moderate cognitive impairment, a significant cohort of older people in general [24, 55, 70, 93]. The ICECAP-O is also preference based which potentially facilitates its use in economic evaluations. Some commentators have suggested that the ICECAP-O is not suitable for use in CUA because of its focus on capabilities which does not enable the calculation of QALYs [24, 94]. However, other commentators have indicated that the ICECAP-O may be used within the framework of economic evaluation, with a revised capability based methodology for capturing the benefits associated with new interventions and/or service innovations [95, 96].

Overall for both the community and residential aged care contexts it’s important to emphasize the breadth of dimensions that affect older people’s quality of life. Compared to the EQ-5D, although the ASCOT and ICECAP-O do not have a health dimension *per se*, they are more sensitive to change and are more associated with broader quality of life beyond health [24]. This review argues that the choice of instrument is determined by the objective of the intervention being assessed; the EQ-5D being preferred for interventions aimed at maintaining health while the ASCOT and ICECAP-O are preferred for interventions with broader benefits beyond health such as service delivery innovations in the aged care sector [24, 49, 55].

A limitation of this study was that due to the heterogeneity of and the lack of adequate data from the studies included in our sample, it was not possible to conduct any meta-correlations or meta-regressions to empirically test whether the instruments used in the studies included in this review perform differently in various contexts.

## Conclusions

This review has highlighted that for older people quality of life is a multi-dimensional concept, being defined by broader dimensions of quality of life in addition to health status. Older people typically derive wider quality of life benefits from service innovations in aged care that may or may not also have a positive impact upon health status. In order to reflect the multi-dimensionality of quality of life and to capture wider quality of life benefits within an economic evaluation framework the most appropriate quality of life instrument for application in the aged care sector is one that ideally measures not only health status and functional ability but also wider quality of life dimensions of importance to older people such as independence, psychological wellbeing, social relationships and social connectedness.

Currently no single instrument exists which is preference based and commensurate with the QALY scale (and therefore appropriate for application in economic evaluation) incorporating both health status and the broader elements of quality of life previously highlighted.

In the absence of a single ideal instrument for CUA to assess the cost effectiveness of service innovations in the aged care sector, this review recommends the use of a generic preference based instrument, the EQ-5D to obtain QALYs in combination with the ICECAP-O or the ASCOT to facilitate the measurement and valuation of broader quality of life benefits as defined by older people.

## Additional file

Additional file 1:
**Medline search strategy.** Database(s): Ovid MEDLINE(R) In-Process & Other Non-Indexed Citations and Ovid MEDLINE(R) 1946 to Present. (PDF 158 kb)

## Abbreviations

15D: 15-Dimensions
AQOL: Assessment of quality of life
ASCOT: Adult Social Care Outcomes Toolkit
CASP-19: Control Autonomy Self-realization and Pleasure
CUA: Cost utility analysis
DCE: Discrete choice experiment
EQ-5D: EuroQol - 5 Dimensions
HRQL: Health related quality of life
HUI2: Health Utilities Index Mark2
HUI3: Health Utilities Index Mark3
ICECAP-O: ICEpop CAPability measure for Older people
NHP: Nottingham Health Profile
OPQOL: Older People’s Quality of Life
QALYs: Quality adjusted life years
QWB: Quality of Wellbeing scale
SF-12: Short Form 12
SF-36: Short Form 36
SF-6D: Short Form 6 Dimensions
SG: Standard gamble
TTO: Time trade off
VAS: Visual analogue scale
WHOQoL-Bref: World Health Organisation Quality of Life brief instrument
WHOQoL-Old: World Health Organisation Quality of Life Instrument-Older Adults Module
