# Supplementary material for: A systematic review of instruments for measuring outcomes in economic evaluation within aged care
Source: Health Qual Life Outcomes. 2015 Nov 9;13:179. doi: 10.1186/s12955-015-0372-8 (PMC4640110; doi:10.1186/s12955-015-0372-8)
Supplement: Additional file 1: — Medline search strategy. Database(s): Ovid MEDLINE(R) In-Process & Other Non-Indexed Citations and Ovid MEDLINE(R) 1946 to Present. (PDF 158 kb) [file 12955_2015_372_MOESM1_ESM.pdf]

Appendix 1: Medline search strategy

Database(s): Ovid MEDLINE(R) In-Process & Other Non-Indexed Citations and Ovid MEDLINE(R) 1946 to Present

# ▲	Searches	Results
1	"Quality of Life"/	129051
2	(QOL or OQOL or HRQOL or HRQL or "quality of life").tw.	171394
3	or/1-2	216698
4	Questionnaires/ or Self report/	334445
5	Health status indicators/	20847
6	(questionnaire? or instrument? or measures or self report? or indices or index or inventory or inventories or tool? or score? or indicator? or scale or scales or rating* or assessment or survey*).tw.	3210785
7	ICECAP-O.tw.	21
8	(SF12 or SF36 or SF6D or ((SF or short form) adj2 ("12" or "36" or 6D))).tw.	22849
9	(EQ5D or EuroQoL or EQ-5D).tw.	5025
10	(ASCOT or WHOQoL OLD or WHO-QoL OLD or "Ferrans and Powers QLI").tw.	367
11	or/4-10	3290096
12	"reproducibility of results"/	298553
13	(reliab* or valid* or reproducib*).tw.	817917
14	Psychometrics/	57939
15	Psychometric*.tw.	29406
16	or/12-15	1014358
17	aged/ or "aged, 80 and over"/ or frail elderly/	2480632
18	(elder* or geriatric* or old age* or ((old* or aged) adj (person or people* or adult* or resident* or population* or men* or women* or male* or female*))).tw.	418495
19	(aged adj ("65" or "70" or "75" or "80" or "85")).tw.	26162
20	or/17-19	2664538
21	(Nursing home* or Long term care or Longterm care or Residential aged	51856

	care or LTCF or Aged care facilit* or care home* or care facilit* or residential care or "institutionalised elders" or "Institutionalised elderly" or institutionalized elder* or skilled nursing facilit*).tw.	
22	((extended care adj2 facilit*) or (geriatric adj2 (home* or facilit* or institution*)) or (long-term care adj2 (facilit* or institution* or setting* or resident* or provider*)) or (LTC adj2 (facilit* or institution* or setting* or resident* or provider*)) or (longterm care adj2 (facilit* or institution* or setting* or resident* or provider*)) or (residential adj2 (home* or care or facilit*)) or (long-stay adj2 (facilit* or institution* or resident*))).tw.	12039
23	Homes for the Aged/ or Health Services for the Aged/ or Nursing Homes/ or intermediate care facilities/ or skilled nursing facilities/ or Long-Term Care/	69051
24	home nursing/ or respite care/ or Home Care Services/ or Social Welfare/ or Community Health Services/ or "Activities of Daily Living"/ or Independent Living/ or Self Care/	144611
25	((community or home* or respite or social or aged or self) adj5 (care* or welfare* or support*)).tw.	127945
26	(((home or community or urban or rural or town or village) adj5 (dwelling or based)) or (living adj5 (home or community or independent*)) or ((retire* or senior*) adj5 (home* or communit* or facilit* or institution* or setting* or context))).tw.	82891
27	or/21-26	370114
28	3 and 11 and 16 and 20 and 27	2382
**29**	**limit 28 to (english language and humans and yr="2000 - 2015" and "all aged (65 and over)")**	**1816**
